# Identification of potential core genes and pathways predicting pathogenesis in head and neck squamous cell carcinoma

**DOI:** 10.1042/BSR20204148

**Published:** 2021-05-27

**Authors:** Mengmeng Wang, Bin Zhong, Man Li, Yanjuan Wang, Huaian Yang, Ke Du

**Affiliations:** 1 School of Pharmacy, Department of Pharmacology, China Medical University, Shenyang, Liaoning 110122, China; 2 Department of Otolaryngology Head and Neck Surgery, the Sleep Medical Center, Shengjing Hospital of China Medical University, Shenyang, Liaoning 110122, China; 3 Department of Respiratory Medicine, The First Affiliated Hospital of Gannan Medical University, Ganzhou, Jiangxi 341000, China; 4 Depatment of Radiology and Medical Imaging, the First Affiliated of JinZhou Medical University, JinZhou 121000, China; 5 School of Pharmacy, Shenyang Pharmaceutical University, 103 Wenhua Road, Shenyang 110016, China

**Keywords:** Differently expressed genes, Head and neck squamous cell carcinoma, Prognosis, Protein-protein interactions network, Survival analysis

## Abstract

Head and neck squamous cell carcinoma (HNSCC) is the most common subtype of head and neck cancer; however, its pathogenesis and potential therapeutic targets remain largely unknown. In the present study, we analyzed three gene expression profiles and screened differentially expressed genes (DEGs) between HNSCC and normal tissues. The DEGs were subjected to gene ontology (GO), Kyoto encyclopedia of genes and genomes (KEGG), protein–protein interaction (PPI), and survival analyses, while the connectivity map (CMap) database was used to predict candidate small molecules that may reverse the biological state of HNSCC. Finally, we measured the expression of the most relevant core gene *in vitro* and examined the effect of the top predicted potential drug against the proliferation of HNSCC cell lines. Among the 208 DEGs and ten hub genes identified, CDK1 and CDC45 were associated with unfavorable HNSCC prognosis, and three potential small molecule drugs for treating HNSCC were identified. Increased CDK1 expression was confirmed in HNSCC cells, and menadione, the top predicted potential drug, exerted significant inhibitory effects against HNSCC cell proliferation and markedly reversed CDK1 expression. Together, the findings of the present study suggest that the ten hub genes and pathways identified may be closely related to HNSCC pathogenesis. In particular, CDK1 and CDC45 overexpression could be reliable biomarkers for predicting unfavorable prognosis in patients with HNSCC, while the new candidate small molecules identified by CMap analysis provide new avenues for the development of potential drugs to treat HNSCC.

## Background

Head and neck cancer is one of the most prevalent malignancies worldwide, with an estimated 600,000 new cases every year [1]. The most common type of head and neck cancer is head and neck squamous cell carcinoma (HNSCC), which is characterized by a high mortality rate due to postoperative metastasis, recurrence, and poor prognosis [2]. Despite various advances in surgery, radiotherapy, and chemotherapy in recent decades, the clinical outcomes of patients with HNSCC remain unchanged [3]. Therefore, it is necessary to identify potential new biomarkers and the pathways and molecular mechanisms underlying carcinogenesis to develop novel diagnostic and treatment strategies for HNSCC.

The molecular pathogenesis of HNSCC is thought to involve a combination of somatic mutations alongside epigenetic and transcriptional alterations. For example, Schmitt et al. found that impaired ryanodine receptor 2 (RYR2) function through either somatic mutation or epigenetic silencing is a common event in HNSCC pathogenesis [4]. In addition, nonsense mutations in protocadherin FAT1 have been found to result in the loss of tumor suppression in HNSCC [5], while Sun et al. reported that HNSCC samples display robust STAT3, PI3K, and AKT expression/activation compared with that in normal squamous epithelium [6]. Moreover, lncRNA HOX transcript antisense intergenic RNA (HOTAIR) polymorphism has been associated with an increased risk of HNSCC [7], and Kong et al. finding that targeting HOTAIR induces mitochondria-related apoptosis and inhibits tumor growth in HNSCC *in vitro* and *in vivo* [8]. However, the majority of studies have focused on a single genetic event or the results of a single cohort study, thus, limiting the scope of their findings. It is, therefore, important to identify differentially expressed genes (DEGs) and study the interactions among them in HNSCC.

Bioinformatics analyses have recently been used to explore the molecular mechanisms of diseases by data mining at the molecular level and have significantly improved our understanding of cancer [9,10]. The Gene Expression Omnibus (GEO) is an international public repository that collects data from different microarray platforms and provides a resource for data mining to uncover molecular variations in a wide variety of tumors, which are important for elucidating the molecular mechanisms underlying tumor pathogenesis and identifying potential biomarkers to improve early diagnosis and prognosis.

In the present study, we screened DEGs between HNSCC and normal tissues using bioinformatics analyses of GEO data and then performed Gene Ontology (GO) functional annotation analysis and Kyoto Encyclopedia of Genes and Genomes (KEGG) pathway enrichment analysis. Ten hub genes related to HNSCC were identified using protein–protein interaction (PPI) network analysis and their effects on survival were determined using Gene Expression Profiling Interactive Analysis (GEPIA). In addition, we identified potential small molecule drugs that could reverse HNSCC-induced gene expression using connectivity map (CMap) database analysis. Finally, we validated the increased expression of the most relevant core gene, CDK1, in various HNSCC cell lines and demonstrated that menadione, the top predicted drug, could inhibit HNSCC cell proliferation and reverse CDK1 expression. Thus, these potential small molecules can serve as potential drug candidates for HNSCC to block CDK1 activity and future studies are warranted to confirm their efficacy.

## Methods

### Data source

The transcriptional profile datasets analyzed in this study were obtained from NCBI GEO databases (https://www.ncbi.nlm.nih.gov/geo/). Three gene expression profiles were selected: GSE29330, GSE59102, and GSE23036. The microarray data from GSE29330 was based on the GPL570 Platform (HG-U133_Plus_2; Affymetrix Human Genome U133 Plus 2.0 Array), GSE59102 was based on the GPL6480 Platform (Agilent-014850; Whole Human Genome Microarray), and GSE23036 was based on the GPL571 Platform (HGU133A_Plus_2; Affymetrix Human Genome U133A Plus 2.0 Array).

### Identification of DEGs

The GEO2R online analysis tool (https://www.ncbi.nlm.nih.gov/geo/geo2r/) was used to identify DEGs between HNSCC and normal samples. Fold-change (FC) and adjusted p-values were calculated, with a |log (FC)| ≥ 1 and an adj. *P*-value < 0.05 defined as the cutoff criteria for DEGs. Overlapping DEGs were identified using the Venn diagram web tool (https://bioinformatics.psb.ugent.be/webtools/Venn/).

### Functional and pathway enrichment analysis

GO provides a framework to describe molecular functions (MF), biological processes (BP), and cellular components (CC), while the KEGG database is widely used to elucidate biological pathways involving DEGs. Both GO annotation and KEGG pathway enrichment analyses were performed using Database for Annotation, Visualization and Integrated Discovery (DAVID) tools (https://david.ncifcrf.gov/). *P*-value < 0.05 and gene counts ≥ 5 were considered statistically significant.

### PPI network analysis and hub gene identification

The evaluate the potential PPIs among the DEGs identified, we used the STRING online database (http://string-db.org/). PPIs between DEG pairs with a combined score of >0.7 (high confidence) were visualized as a network using Cytoscape software (www.cytoscape.org/), in which highly intra-connected nodes tend to be more important for maintaining the stability of the gene expression network. In addition, we used the Cytoscape CytoHubba plug-in to obtain the degree of each protein node and identify the top ten most connected hub genes.

### Hub gene expression and survival analysis

The Cancer Genome Atlas (TCGA) data visualization web-tool, GEPIA (http://gepia.cancerpku.cn), interactively analyzes cancer-related and normal genes [11]. In the present study, we used GEPIA for expression and survival analysis to evaluate the prognostic value of the identified hub genes in patients with HNSCC. Hub genes that were significantly associated with survival rate (log rank *P* value < 0.05) were considered to be key genes for HNSCC prognosis.

### Screening of potential small molecule drugs

CMap (https://portals.broadinstitute.org/cmap/) is a collection of genome-wide transcriptional expression data that enables the discovery of functional connections between drugs, genes, and diseases via transient changes in common gene expression [12]. First, we divided the DEGs obtained using the Venn package into an up-regulated group and a down-regulated group. To predict potential small molecule drugs that might reverse gene expression in HNSCC, the DEGs were uploaded into CMap for gene set enrichment analysis (GSEA): those with enrichment values close to -1 were deemed more likely to reverse the biological state of HNSCC. The results were ranked according to their negative connectivity value (close to -1) and those with a *P*-value < 0.05 were considered statistically significant. The 2D chemical structures of the top three small molecules were determined using SciFinder (https://scifinder.cas.org/scifinder/view/scifinder/scifinderExplore.jsf), a research discovery application that provides integrated access to the source of references, substances, and reactions in chemistry and related sciences.

### Cell lines and culture conditions

Three HNSCC cell lines (CAL27, FaDu, and SCC25) and one normal human oral keratinocyte (HOK) cell line were used in the present study. CAL27 and SCC25 cells were obtained from the ATCC. FaDu cells were obtained from the Cell Bank of the Chinese Academy of Sciences. HOK cells were purchased from ScienCell Research Laboratories. All cell lines were cultured in DMEM (HyClone), supplemented with 10% fetal bovine serum (FBS; TBD, Tianjin, China), 1% glutamine, and 1% penicillin–streptomycin in 5% CO_2_ at 37°C.

### Real-time PCR assay

Total RNA was isolated from cultured cells using TRIzol reagent (CWBIO), according to the manufacturer’s instructions. Complementary DNA (cDNA) was synthesized using a PrimeScript RT-PCR Kit (Takara, Dalian, China) primed with oligo (dT). Quantitative reverse-transcription PCR was carried out using an SYBR Green PCR Mix Kit (Takara) with the following primer sequences: CDK1, 5′-CAGTCTTCAGGATGTGCTTAT-3′ (forward) and 5′-TGACCAGGAGGGATAGAAT-3′ (reverse); and GAPDH, 5′-CTGCACCACCAACTGCTTAG-3′ (forward) and 5′-AGGTCCACCACTGACACGTT-3′ (reverse). Relative mRNA levels were quantified using the internal control gene, GAPDH.

### Western blot assay

Protein extraction and western blotting were performed as described previously [8]. Membranes were probed with primary rabbit antibodies against CDK1 (1:1000, Abcam) and β-actin (1:1000, SantaCruz), followed by goat anti-rabbit secondary antibodies conjugated to horseradish peroxidase (1:2000; Santa Cruz). Immunoreactive bands were visualized using an enhanced chemiluminescent kit (ECL+, Amersham Biosciences) and measured using QualityOne analysis software (BioRad).

### Cell proliferation assay

Cell proliferation was detected using a CCK-8 assay (Dojindo, Japan). Briefly, cells were seeded in 96-well plates (3000 cells/well), cultured in DMEM for 24 h and treated with 10 μM menadione (MedChemExpress) for 0, 12, 24, or 48 h. After incubation, the cells were treated with CCK-8 reagents according to the manufacturer’s instructions and absorbance was measured at 450 nm using a multi-mode reader (LD942, Beijing, China). All assays were performed in triplicate.

## Results

### DEG identification

**Figure 1:**
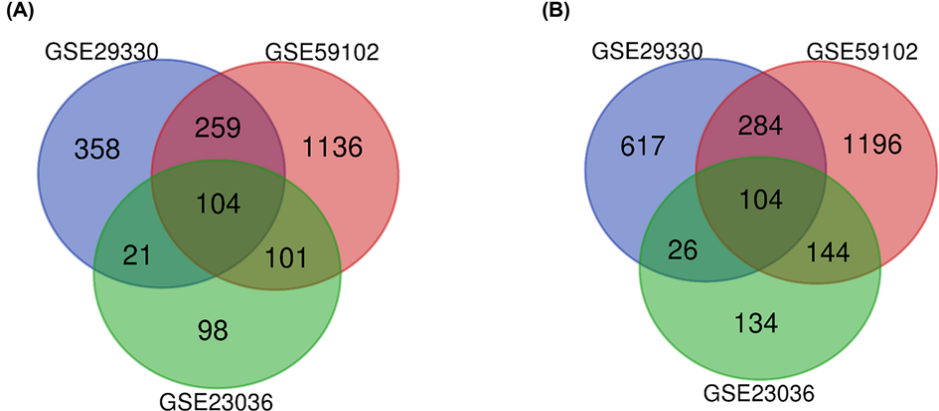
Venn diagram of differentially expressed genes (DEGs) common to all three GEO datasets (**A**) Up-regulated genes; (**B**) Down-regulated genes.

To identify DEGs in HNSCC, we utilized three gene expression profiles: GSE29330, GSE59102, and GSE23036. GSE29330 included 13 primary HNSCC tumor samples and 5 normal mucosa samples from control patients without cancer. GSE59102 contained 29 HNSCC samples and 13 marginal samples, while GSE23036 contained 63 HNSCC biopsy samples and 5 mucosa samples (Table 1). From these data, we obtained a total of 4582 DEGs, including 2077 up-regulated genes and 2505 down-regulated genes. In addition, the Venn diagram web-tool was used to intersect the three sets of DEGs and identify 208 that overlapped, of which 104 were significantly up-regulated and 104 were down-regulated (Figure 1A,B).

**Table 1:** Sample composition of the three microarray datasets derived from the GEO database

Dataset ID	HNSCC samples	Normal samples	Total samples
GSE29330	13	5	18
GSE59102	29	13	42
GSE23036	63	5	68

Abbreviations: GEO, Gene Expression Omnibus; HNSCC, head and neck squamous cell carcinoma.

### DEG enrichment analysis

To elucidate the biological functions of the 208 DEGs identified, we used DAVID to conduct GO functional and KEGG pathway enrichment analyses. GO analysis indicated that the DEGs were mainly enriched in BPs including M phase, mitotic cell cycle, epidermis development, nuclear division, and mitosis, as well as CCs such as collagen, extracellular matrix (ECM), and the cytoskeleton. In addition, KEGG pathway analysis indicated that the most enriched pathways were the ECM–receptor interaction, focal adhesion, cell cycle, and pathways in cancer (Table 2).

**Table 2:** DEG functional and pathway enrichment analysis

Category	Term	Description	Count	*P*-value
BP	GO:0000279	M phase	16	0.0350954
BP	GO:0000278	Mitotic cell cycle	17	0.0354634
BP	GO:0022403	Cell cycle phase	18	0.0377504
BP	GO:0008544	Epidermis development	12	0.040066
BP	GO:0000280	Nuclear division	13	0.0426893
BP	GO:0007067	Mitosis	13	0.0426893
CC	GO:0005581	Collagen	7	0.0034622
CC	GO:0005578	Proteinaceous extracellular matrix	17	0.0011671
CC	GO:0031012	Extracellular matrix	17	0.0031239
CC	GO:0044421	Extracellular region part	29	0.0058089
CC	GO:0044420	Extracellular matrix part	10	0.0108147
CC	GO:0005856	Cytoskeleton	34	0.0482425
KEGG pathway	hsa04512	ECM–receptor interaction	9	1.46E-05
KEGG pathway	hsa04510	Focal adhesion	9	0.0054566
KEGG pathway	hsa04110	Cell cycle	6	0.0266123
KEGG pathway	hsa05200	Pathways in cancer	10	0.0318641

Abbreviations: BP, biological process; CC, cellular component; DEG, differentially expressed gene; GO, Gene Ontology; KEGG, Kyoto Encyclopedia of Genes and Genomes.

### PPI network construction and hub gene identification

**Figure 2:**
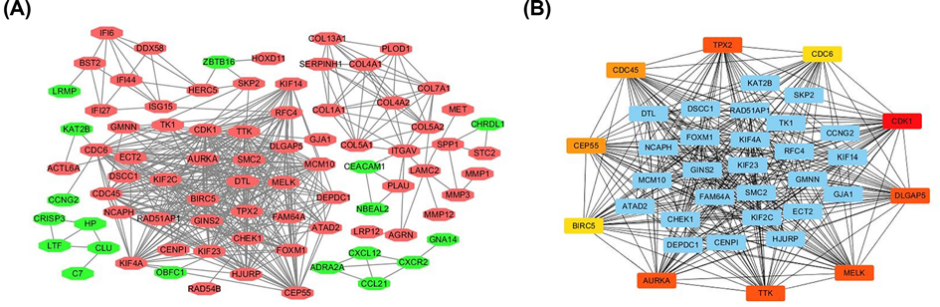
Protein–protein interaction (PPI) network constructed with the differentially expressed genes (DEGs) (**A**) Visualized PPI analysis of DEGs. Red nodes represent up-regulated genes, and green nodes represent down-regulated genes. (**B**) The top 10 genes in the PPI network.

**Figure 3:**
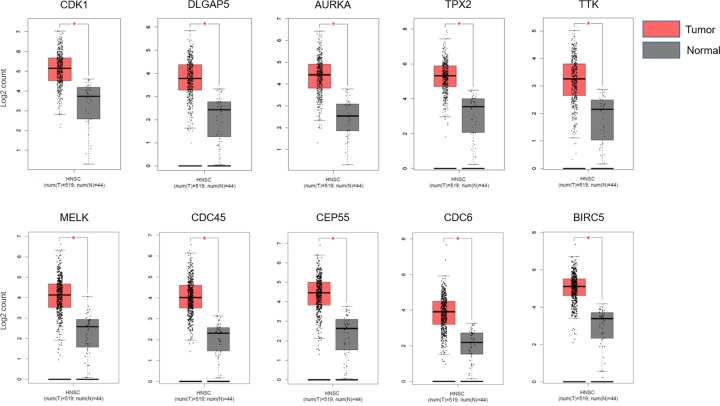
Expression of the ten hub genes in tumor and normal tissues Expression values of genes are log2-transformed.

Next, we analyzed PPIs between overlapping DEGs using STRING, identifying a network of relevant PPIs consisting of 207 nodes and 454 edges that were visualized using Cytoscape (Figure 2A,B). Based on their degree of connectivity within the PPI network, the top ten hub genes were selected: CDK1, DLGAP5, AURKA, TPX2, TTK, MELK, CDC45, CEP55, CDC6, and DIRC5 (Table 3), all of which were up-regulated in GEO. GEPIA of hub gene expression in HNSCC revealed that the expression of all ten hub genes was significantly increased in 519 HNSCC tissues compared with that in normal control tissues (*P*<0.05, Figure 3).

**Table 3:** Top ten hub genes with higher degree of connectivity

Gene symbol	Gene description	Degree	Regulation
CDK1	Cyclin dependent kinase 1	33	Up
DLGAP5	DLG-associated protein 5	28	Up
AURKA	Aurora kinase A	28	Up
TPX2	TPX2 microtubule nucleation factor	28	Up
TTK	TTK protein kinase	28	Up
MELK	Maternal embryonic leucine zipper kinase	28	Up
CDC45	Cell division cycle 45	27	Up
CEP55	Centrosomal protein 55	27	Up
CDC6	Cell division cycle 6	26	Up
BIRC5	Baculoviral IAP repeat containing 5	26	Up

### Hub gene survival analysis

**Figure 4:**
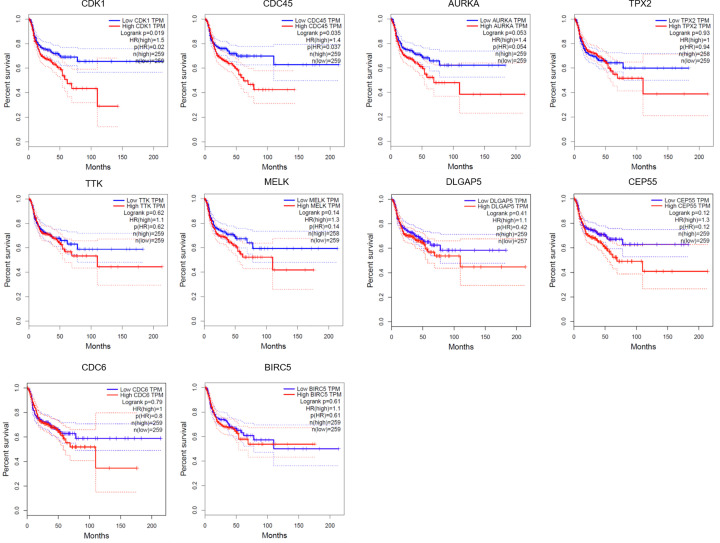
Survival analysis for the top ten hub genes expressed in HNSCC patients

To investigate the prognostic value of the ten hub genes in HNSCC, we used GEPIA. High CDK1 and CDC45 expression were associated with unfavorable relapse-free survival in patients with HNSCC (*P*<0.05, Figure 4). Similarly, patients with high expression of the other eight hub genes also had a tendency toward unfavorable survival compared with that in patients showing low gene expression; however, this correlation was not significant (*P*>0.05, Figure 4). Therefore, CDK1 and CDC45 were identified as unfavorable prognostic factors in patients with HNSCC.

### *In vitro* validation of CDK1 overexpression in HNSCC cell lines

**Figure 5:**
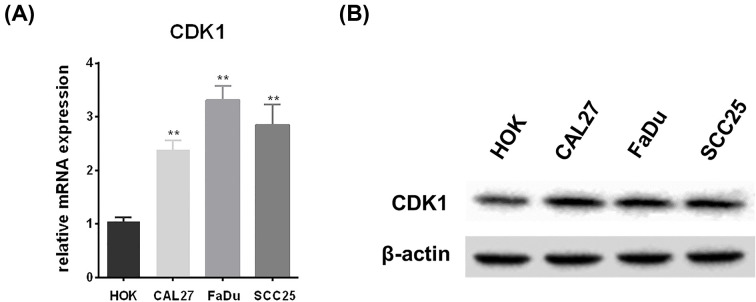
*In vitro* validation of CDK1 as over-expressed gene in HNSCC cell lines (**A**) Estimation of CDK1 mRNA levels in HKO, CAL27, FaDu and SCC25 cell lines by Real-time PCR. (**B**) The protein expression of CDK1 was analyzed by Western blotting in HKO, CAL27, FaDu, and SCC25 cell lines; *P<0.05, **P<0.01 versus vehicle-treated cells.

To validate CDK1 as an overexpressed core gene *in vitro*, we investigated its expression levels in the three HNSCC cell lines and the normal HOK cell line. Interestingly, higher CDK1 mRNA and protein expression were detected in the HNSCC cell lines (Figure 5A,B), consistent with our observation that CDK1 expression is significantly increased in HNSCC compared with that in normal control tissues.

### Screening of potential small molecule drugs

**Figure 6:**
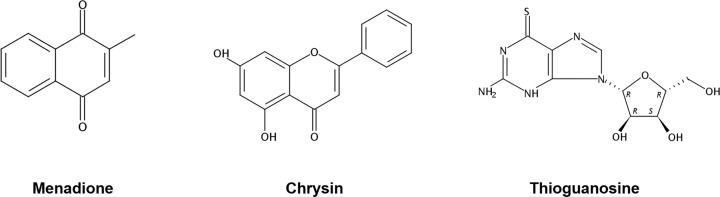
Chemical structure of three potential small molecule drugs

To predict candidate small molecules for treating HNSCC, we uploaded the up- and downregulated DEGs to the CMap database for GSEA. The top three small molecules (menadione, chrysin, and thioguanosine) with satisfactory enrichment scores are listed in Table 4 and their chemical structures are shown in Figure 6. These small molecules were the most likely to reverse HNSCC-related gene expression and could help to develop new targeted drugs to treat HNSCC.

**Table 4:** Three most significant small molecule drugs

Rank	CMap name	Mean	N	Enrichment	*P*-value	CAS registry Number
1	Menadione	-0.745	2	-0.937	0.00847	58-27-5
2	Chrysin	-0.696	3	-0.886	0.00288	480-40-0
3	Thioguanosine	-0.663	4	-0.876	0.00052	85-31-4

CMap, connectivity map.

### Correlation between the inhibition of HNSCC cell proliferation by menadione and CDK1 expression

**Figure 7:**
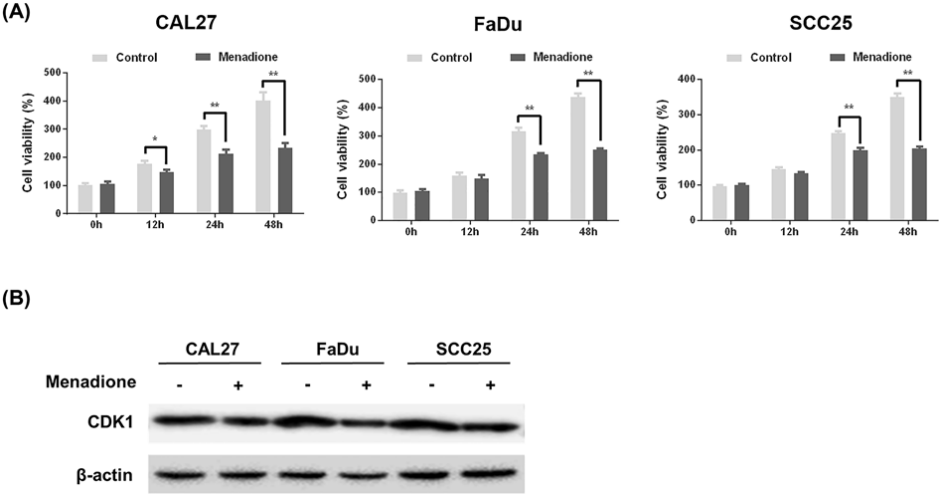
Correlation of the inhibitory effect of menadione on HNSCC cell proliferation with CDK1 expression (**A**) Estimation of cell viability in CAL27, FaDu, and SCC25 cell lines treated with 10 μM menadione or distilled water for 0, 12, 24, 48 h by CCK-8 assays. (**B**) Cells were treated with 10 μM Menadione or distilled water for 24 h. The expression of CDK1 was analyzed by Western blotting; *P<0.05, **P<0.01 versus vehicle-treated cells.

To determine the value of these potential small molecule drugs for treating HNSCC, we examined the effect of the top predicted potential drug, menadione, on the proliferation of HNSCC cell lines. We found that the proliferation of all three HNSCC cell lines, as measured using a CCK-8 assay, was markedly inhibited after 24 h of exposure to menadione (Figure 7A). Next, we explored whether treatment with menadione could reverse gene expression in HNSCC by measuring CDK1 protein expression using Western blot assays. Menadione dramatically inhibited the expression of CDK1, which is overexpressed in HNSCC (Figure 7B), suggesting that the inhibitory effect of menadione against HNSCC cell proliferation could be related to its inhibition of CDK1 overexpression.

## Discussion

HNSCC is one of the most prevalent types of cancer worldwide and is associated with a poor prognosis [1] due to the lack of specific therapeutic targets, which currently limits standard therapies to surgery, chemotherapy, and radiotherapy [2]. Therefore, it is crucial to identify more effective therapeutic targets for HNSCC.

In this study, we identified 104 up-regulated DEGs and 104 down-regulated DEGs based on HNSCC gene expression profiling data from the GEO database. These genes were associated with GO BP terms such as M phase, mitotic cell cycle, epidermis development, nuclear division, and mitosis, as well as KEGG terms such as ECM–receptor interaction, focal adhesion, cell cycle, and pathways in cancer. To determine the relationships between the DEGs, we constructed a PPI network and identified ten hub genes: CDK1, DLGAP5, AURKA, TPX2, TTK, MELK, CDC45, CEP55, CDC6, and DIRC5. All of these genes were up-regulated in HNSCC and were also verified as significantly highly expressed in patients with HNSCC based on GEPIA analysis. In addition, we found that CDK1 and CDC45 overexpression were unfavorable prognostic factors in patients with HNSCC and validated CDK1 overexpression *in vitro*. Furthermore, we found that the top predicted potential drug, menadione, exerted significant inhibitory effects against HNSCC cell proliferation and markedly reversed CDK1 expression.

Functional and pathway enrichment analysis revealed that the DEGs identified in the present study were mainly associated with the cell cycle, mitosis, nuclear division, pathways in cancer, ECM–receptor interactions, and focal adhesion. These findings are consistent with the knowledge that processes regulating the cell cycle, mitosis, cell adhesion, and ECM interactions are all closely related to mechanisms of tumorigenesis, invasion, and metastasis [13–15]. Various studies have shown that the mitotic cell cycle, cell adhesion, and ECM interactions also play important roles in HNSCC occurrence and progression. For example, Prystowsky et al. found that the histone deacetylase inhibitor LBH589 suppresses the expression of mitotic genes, causing G2/M arrest and death in HNSCC cell lines [16]. Similarly, Göttgens et al. reported that the mitotic inhibitor palbociclib could improve the radiosensitivity of HNSCC cells [17], while Li et al. showed that chemokine receptor 7 (CCR7) could regulate the adhesion and migration of metastatic HNSCC cells and serve as a potential therapeutic target for HNSCC [18]. Furthermore, Koshizuka et al. found that the restoration of miR-1 and miR-206 significantly inhibited the aggressiveness of HNSCC cells, which may be related to the regulation of the focal adhesion and ECM–receptor interaction pathways [19]. Moreover, focal adhesion kinase (FAK) overexpression and activation have been detected in multiple tumor types, including HNSCC, and FAK inhibition has been shown to induce cell cycle arrest and apoptosis and significantly decrease cell growth, invasion, and migration in HNSCC cell lines [20]. Therefore, further studies of these signaling pathways could improve our understanding of the molecular pathogenesis of HNSCC.

Cyclin-dependent kinase 1 (CDK1) plays an important role in the transition from G2 to M phase during the cell cycle [21,22], and multiple studies have verified that CDK1 acts as an oncogene. Indeed, CDK1 overexpression has been demonstrated in various tumors, including melanoma [23], pancreatic cancer [24], colon cancer [25,26], and mammary carcinoma [27,28]. In addition, some of these studies have shown that high CDK1 expression may be associated with poor prognosis in these malignancies. Uddin et al. found that CDK1 is a prognostic marker for colon cancer [26], while Piao et al. showed that CDK1 may play a role in pancreatic ductal adenocarcinoma (PDAC) progression and could be a prognostic biomarker for patients with PDAC [24]. Moreover, Liu et al. reported that high CDK1 expression is closely associated with poor clinical prognosis in breast cancer [28]. In HNSCC, some studies have demonstrated that high CDK1 expression at both the mRNA and protein levels may occur during the early stages of carcinogenesis [29], and that CDK1 overexpression is related to tumorigenesis [30] and malignant transformation [31]. Furthermore, some research has suggested that CDK1 is related to anti-cancer activity and enhanced sensitivity to radiotherapy and anti-cancer drugs in HNSCC cells [32,33]. Together, these previous results support our findings that CDK1 was highly expressed in HNSCC both *in vivo* and *in vitro*, and that high CDK1 expression is associated with the unfavorable relapse-free survival of patients with HNSCC. Consequently, we suggest that CDK1 may be a novel predictive factor for poor prognosis in HNSCC.

The second key gene identified in our study, cell division cycle 45 (CDC45), is a component of the CDC45 mini-chromosome maintenance protein complex (MCM) and the GINS (CMG) helicase complex which is required for DNA synthesis and genome stability during genome duplication [34]. Studies have shown that CDC45 is a proliferation-associated antigen that may promote tumorigenesis and metastasis [35,36], and that CDC45 is overexpressed in several cancer-derived cell lines, including carcinoma-, sarcoma-, leukemia-, and lymphoma-derived cells [35]. Moreover, CDC45 has previously been identified as a candidate oncogene in non-small cell lung cancer [37], hepatocellular carcinoma [38], papillary thyroid cancer [39], and cervical cancer [40]; however, the expression profile and function of CDC45 in HNSCC have rarely been reported. In the present study, we identified CDC45 as a hub gene that was up-regulated in HNSCC tissues and associated with an adverse prognosis in patients with HNSCC. Therefore, we suggest that CDC45 could serve as a novel therapeutic target and poor prognostic factor in HNSCC.

Finally, we also screened a set of candidate drugs that were most likely to reverse abnormal gene expression in HNSCC using the CMap database. Menadione, the top predicted molecule against HNSCC, is a synthetic form of vitamin K that is important for blood clotting and bone formation [41,42] and has been reported to induce apoptosis and growth inhibition in multiple tumor cell types, including lung cancer [43], prostate carcinoma [44], breast carcinoma [45], and hepatocellular carcinoma cells [46]. The mechanisms underlying these anti-cancer effects have been associated with the down-regulation of the Wnt pathway [47], oxidative stress [48], mitochondrial dysfunction [49], glycolysis inhibition [50], and cell cycle blockade [51], all of which ultimately lead to apoptotic cell death or growth inhibition. However, to our knowledge, no studies have yet investigated the effect of menadione in HNSCC. We found that menadione markedly inhibited the proliferation of various HNSCC cell lines as well as CDK1 expression. Therefore, we speculate that the molecular mechanism underlying the anti-proliferative effects of menadione may be related to CDK1-dependent cell cycle progression.

In summary, we screened 208 DEGs between HNSCC and adjacent normal tissues using bioinformatic analysis and identified ten key hub genes and important pathways that may play important roles in HNSCC pathogenesis. In particular, CDK1 and CDC45 overexpression were associated with a poor prognosis in HNSCC patients and may therefore be novel reliable biomarkers for diagnosis, survival, and prognosis in HNSCC. Furthermore, in the present study, we demonstrated that menadione could be a new and effective candidate drug for treating HNSCC and, thus, suggest a novel method for screening and developing potential drugs against HNSCC.

## Web links and URLs

Gene expression omnibus (GEO): https://www.ncbi.nlm.nih.gov/geo/. Ensemble: http://asia.ensembl.org/index.html. Database for Annotation, Visualization, and Integration Discovery (DAVID): https://david.ncifcrf.gov/. STRING: https://string-db.org/cgi/input.pl. Cytoscape: https://cytoscape.org/. Venn diagram web-tool: http://bioinformatics.psb.ugent.be/webtools/Venn/. GEPIA: http://gepia.cancerpku.cn. Cmap: https://portals.broadinstitute.org/cmap/.

## Data Availability

The datasets generated and/or analyzed during this study are available from the gene expression omnibus database.

## Abbreviations

BP: biological process
CC: cellular component
CMap: connectivity map
DEG: differentially expressed gene
GEO: gene expression omnibus
GEPIA: gene expression profiling interactive analysis
GO: gene ontology
HNSCC: head and neck squamous cell carcinoma
KEGG: Kyoto encyclopedia of genes and genomes
PPI: protein–protein interaction
TCGA: The Cancer Genome Atlas
